# Pickleball eye injuries: ocular protection recommendations and guidelines

**DOI:** 10.1038/s41433-023-02870-9

**Published:** 2023-12-14

**Authors:** Ethan Waisberg, Joshua Ong, Andrew G. Lee

**Affiliations:** 1 Department of Ophthalmology, University of Cambridge, Cambridge, UK; 2 Department of Ophthalmology and Visual Sciences, University of Michigan Kellogg Eye Center, Ann Arbor, MI USA; 3 Center for Space Medicine, Baylor College of Medicine, Houston, TX USA; 4 Department of Ophthalmology, Blanton Eye Institute, Houston Methodist Hospital, Houston, TX USA; 5 The Houston Methodist Research Institute, Houston Methodist Hospital, Houston, TX USA; 6 Departments of Ophthalmology, Neurology, and Neurosurgery, Weill Cornell Medicine, New York, NY USA; 7 Department of Ophthalmology, University of Texas Medical Branch, Galveston, TX USA; 8 University of Texas MD Anderson Cancer Center, Houston, TX USA; 9 Texas A&M College of Medicine, Bryan, TX USA; 10 Department of Ophthalmology, The University of Iowa Hospitals and Clinics, Iowa City, IA USA

**Keywords:** Public health, Health care, Risk factors

**Fig. 1:**
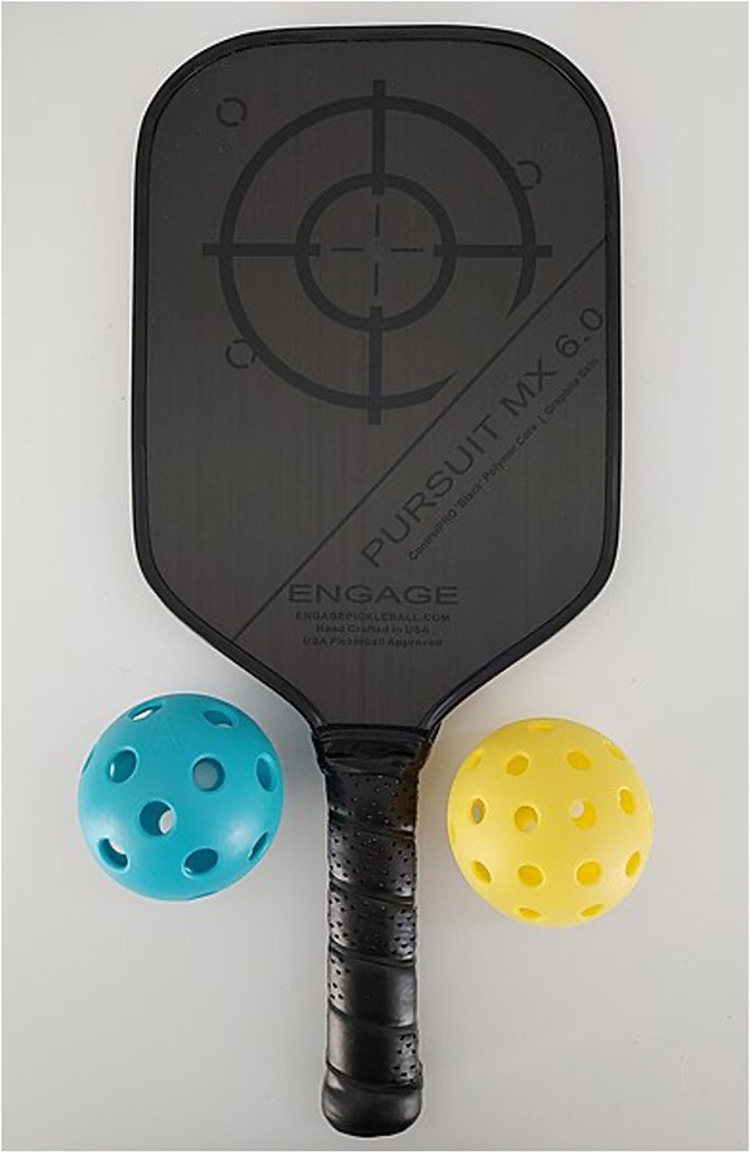
Pickleballs and a pickleball paddle. Pickleballs are typically made of plastic that can move at high velocities. Pickleball paddles are comprised of different materials, including fiberglass or graphite, which may cause traumatic ocular injuries between teammates given the tight proximity in the game. Figure reprinted with permission from OvertAnalyzer in Wikimedia Commons under Creative Commons Attribution-ShareAlike 4.0 International (CC BY-SA 4.0) license (https://creativecommons.org/licenses/by-sa/4.0/legalcode.en).

**Fig. 2:**
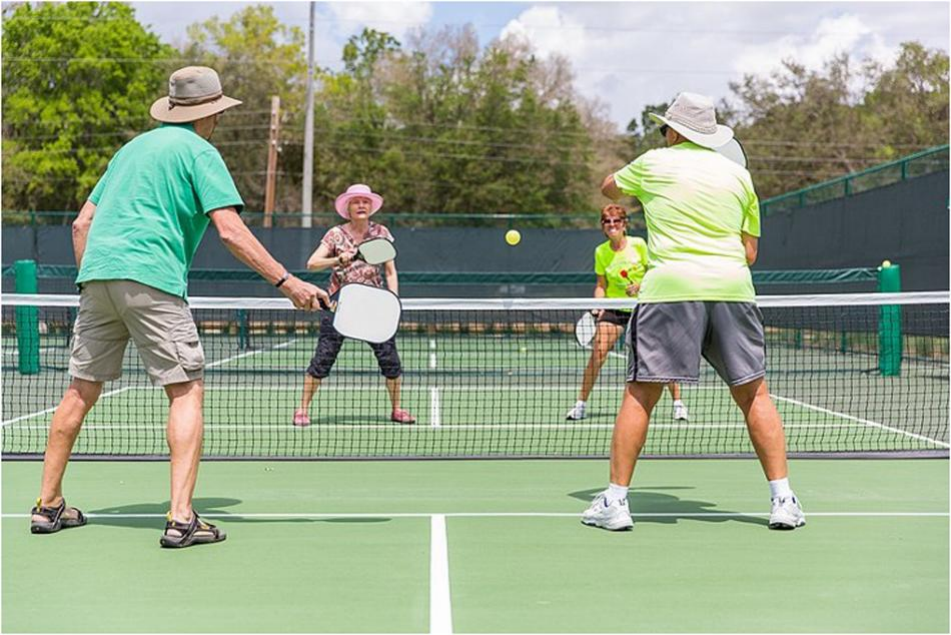
Four individuals playing pickleball. The game is fast paced and in relative close contact to the opposing team, thus, highlighting the need for further insights into proper ocular protection. Pickleball has gained popularity in many demographics, including individuals over the age of 65. Figure reprinted with permission from TheVillagesFL in Wikimedia Commons under Creative Commons Attribution-ShareAlike 4.0 International (CC BY-SA 4.0) license (https://creativecommons.org/licenses/by-sa/4.0/legalcode.en).

Pickleball is one of the fastest growing sports worldwide. According to USA Pickleball and the 2023 Sports & Fitness Industry Association (SFIA), pickleball participation in the United States has increased by 158.6% in the past 3 years [1]. In this newly popular racket sport, a perforated plastic ball is used, and is hit with a composite (often made of fiberglass) or graphite paddle back and forth over a net, while the court is approximately half the size of a tennis court (Fig. 1). The high velocity of pickleball and small court poses a significant risk for eye injuries. Despite the sport’s recent growth in popularity, there is a lack of papers examining the potential risk of injury. Pickleball is also particularly popular in individuals greater than 65-years-old in age, who are particularly vulnerable to eye injuries (Fig. 2). It is critical to evaluate the ocular risks and prevention tactics in this sport, particularly as there is minimal literature on the topic given its rapid popularity. We outline the various ophthalmic risks of pickleball and propose strategies to mitigate these risks.

Pickleball requires a combination of hand-eye coordination, agility and stamina. Additionally, vision plays an essential role in performance in the sport, including maintaining high levels of: visual acuity, dynamic visual acuity and contrast sensitivity. High levels of static visual acuity [2] are required to visualize the court and surroundings. Dynamic visual acuity [3] is required to track a constantly moving pickleball and visualize the opposing players. Contrast sensitivity [4] is required to visualize the pickleball and to make accurate line calls. Without maintaining high levels of these visual parameters, pickleball performance will decrease (Table 1).

**Table 1:** Summary of the currently reported pickleball eye injuries.

Author (ref.)	Ocular injury
Dang [5]	Corneal abrasion, iritis.
Huang [7]	Two cases of traumatic lens subluxation.
Atkinson [6]	One case of a mild vitreous hemorrhage, symptomatic retinal tear and detachment. One case of symptomatic retinal tear and posterior vitreous detachment.

Dang et al. [5] presented the case of a pickleball that bounced off a man’s paddle into his eye, which resulted in a large corneal abrasion. This was the first recorded case of a pickleball causing an anterior segment eye injury. Atkinson et al. [6] presented the first case series of two instances of retinal tears due to pickleball. In the first case, a 66-year-old male experienced mild vitreous hemorrhage and a localized retinal detachment, after being directly hit in the eye by a pickleball [6]. In the second case, a 60-year-old female was struck in the eye by a pickleball, and had a posterior vitreous detachment [6]. No eye protection was worn in both of these cases. Huang and Greven [7] presented two cases of traumatic lens subluxation resulting from pickleball injuries. In one of these cases, following scleral fixation and lens insertion, the patient experienced traumatic glaucoma and postoperative cystoid macular edema.

As participation in pickleball increases, increasing levels of injuries, including ophthalmic injuries are being seen. At the time of writing, no protective eyewear is mandated to play pickleball at any level. Meanwhile similar racket sports like squash and racquetball, have mandatory eyewear rules across various levels of the game. Wearing protective eyewear can significantly reduce eye injury risk by preventing any object from coming into contact with the eye [8]. Certain eyewear can also pose as an advantage by improving visibility by reducing glare with polarized lenses, or include prescription lenses to correct refractive errors. Eyewear should also ideally be wrap-around style to provide maximum levels of ocular protection. Plastic lenses would be ideal to minimize the risk of shattering on impact from a pickleball. Protective eyewear should particularly be considered in individuals at higher risk of retinal detachment, such as individuals with a family history of retinal detachment, aged between 60 and 70 years, high myopia or being pseudophakic [6].

It is also important to consider that pickleball eye injuries are likely underreported, and may not always present to emergency departments. Better surveillance of pickleball eye injuries and understanding of the eye injury risk is required. All things considered, while pickleball is generally considered to be a safe sport due to its non-contact nature, the risk of eye injuries cannot be overlooked.
